# Supplementary material for: Effect of Bis (2-Aminoethyl) Adipamide/Adipic Acid Segment on Polyamide 6: Crystallization Kinetics Study
Source: Polymers (Basel). 2020 May 6;12(5):1067. doi: 10.3390/polym12051067 (PMC7284434; doi:10.3390/polym12051067)
Supplement: Supplementary file 1 [file polymers-12-01067-s001.pdf]

Effect of Bis (2-Aminoethyl) Adipamide/Adipic Acid Segment on Polyamide 6: Crystallization Kinetics Study

Yu-Hao Chen, Palraj Ranganathan, Chin-Wen Chen, Yi-Huan Lee and Syang-Peng Rwei

S1. Synthesis of PA6(BAEA/AA) Copolyamides

The preparing method of bis(2-aminoethyl)adipamide (BAEA) diamine and its organic salt (BAEA/AA salt) has been disclosed in previous work [1–3]. Weighed quantities of ε-caprolactam, BAEA/AA salt, DI water (5.0 wt%), and orthophosphoric acid (0.2 wt%) were added to a 2 L autoclave with a stirrer and vacuum system. After the oxygen in the autoclave had been removed by repeated purging with pure nitrogen under pressure, the autoclave was heated to 200 °C for 1 h under nitrogen at a pressure of 2 kg cm^{-2}, and further heated to 250 °C for 2.0 to 2.5 h in the flowing nitrogen while the pressure was reduced to atmospheric pressure. Low-pressure conditions were maintained until the required melting viscosity was reached. A PA6 (BAEA/AA) was then removed from the bottom duct lid of the 2L reactor and quenched in a water bath. It was subsequently purified by hot water extraction and dried copolyamide particles analysis. As shown in Figure S1, ^{1}H NMR (CF_3COOD, 300 MHz):δ3.83 (C$\underline{\mathbf{H}}_2$NHCO from BAEA/AA segment), δ3.63 (C$\underline{\mathbf{H}}_2$NHCO from PA6 segment), δ2.80 (NHCOC$\underline{\mathbf{H}}_2$ from PA6 segment), δ2.73 (NHCOC$\underline{\mathbf{H}}_2$ from BAEA/AA segment), δ1.95-1.57 (C$\underline{\mathbf{H}}_2$). FT-IR: 3299 (N-H stretching), 2932 (asymmetric stretching of CH_2), 2854 (symmetric stretching of CH_2), 1635 (amide I), 1536 (amide II), and 1262 (amide III) cm^{-1}.

Table S1. Composition and relative viscosity of the PA6 and PA6/(BAEA/AA) copolyamides.

Sample	PA6/(BAEA/AA)/mole%		Relative Viscosity (η_r) [b]
	Feed Ratio	Calculated Ratio [a]	
**PA6**	100/0	100/0	2.74
**PA6-5**	95/5	95.9/4.1	2.50
**PA6-10**	90/10	89.5/9.5	2.46
**PA6-15**	85/15	85.0/15.0	2.29

[a] Determined by ^{1}H NMR in d-TFA solvent based on the integration. [b] Relative viscosity (η_r) measured using an Ubbelohde viscometer in accordance with ASTM-D789. C = 0.1 g dL^{-1} in formic acid (90%) solution.

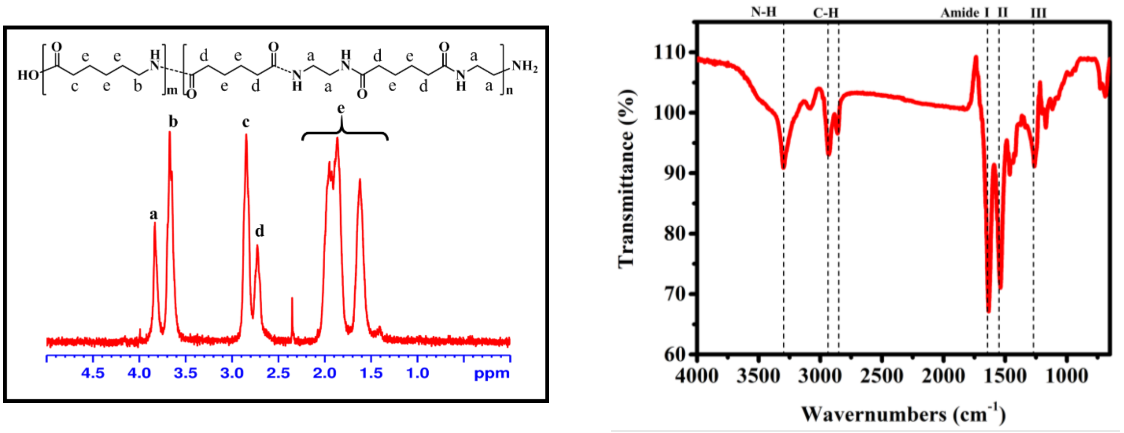

Figure S1. ^{1}H NMR (**left**) and FT-IR (**right**) spectra of PA6-10 (CF_3COOD, 300 MHz).

Table S2. Thermal properties of PA6 and PA6(BAEA/AA) copolyamides at non-isothermal conditions.

Samples	T_o [a] °C	T_p [a] °C	T_f [a] °C	ΔH_c [a] J g^{-1}	T_m [a] °C	ΔH_m [a] J g^{-1}	X_c % [b] %	T_g [c] °C
**PA6**	192	188.9	185	55.2	219.6	47.3	24.9	61.3
**PA6-5**	148.8	143.5	138.2	35.3	186.0	29.9	15.7	44.1
**PA6-10**	115.8	104.8	93.6	31.4	155.8	23.1	12.2	40.3
**PA6-15**	151.6	144.9	136.4	25.2	190.5	20.1	10.6	41.6

[a] the thermal properties are ascertained from the DSC analysis at the non-isothermal condition at a cooling rate of 10 °C min^{-1}, where the T_o, T_p, and T_f represented the onset, peak, and finish crystallization temperature. [b] the X_c% is crystallinity and calculated from $\Delta H_m/\Delta H_m^o$, where ΔH_m^o is the fusion enthalpy of 100% crystalline PA6, which is 190 J g^{-1}. [c] the T_g is measured by the dynamic mechanical analyzer (DMA, Tech Max DMS 6100, Tokyo, Japan), as shown in Figure S2.

Table S3. Thermal properties and the calculated crystallization degrees of PA6 and PA6(BAEA/AA) copolyamides.

Samples	T_c °C	$T_{m\text{-}I}$ [a] °C	$T_{m\text{-}II}$ [a] °C	$T_{m\text{-}III}$ [a] °C	ΔH_m [a] J g^{-1}	X_c % %
	180	212.8	N/A	225.2	44.6	23.5
	185	214.8	N/A	225.1	41.6	21.9
**PA6**	190	217.2	N/A	224.8	38.1	20.1
	195	222.7	N/A	N/A	34.6	18.2
	200	223.6	N/A	N/A	27.7	14.6
	135	169.8	N/A	187.9	27.3	14.4
	140	172.9	N/A	188.3	26.3	13.8
**PA6-5**	145	176.4	N/A	188.8	21.4	11.3
	150	179.9	N/A	189.3	19.9	10.5
	155	184.2	N/A	N/A	11.5	6.1
	95	141.4	164.3	180.4	29.5	15.5
	100	143.2	165.0	180.5	28	14.7
**PA6-10**	105	145.7	165.4	179.6	27.1	14.3
	110	148.9	165.9	179.3	25.3	13.3
	115	152.4	166.8	178.9	22.4	11.8
	135	N/A	191.7	N/A	21.4	11.3
	140	N/A	192.1	N/A	19.4	10.2
**PA6-15**	145	N/A	192.5	N/A	17.8	9.4
	150	N/A	192.9	N/A	15.4	8.1
	155	N/A	193.3	N/A	12.3	6.5

[a] The thermal properties are ascertained from the DSC analysis at the different isothermal conditions.

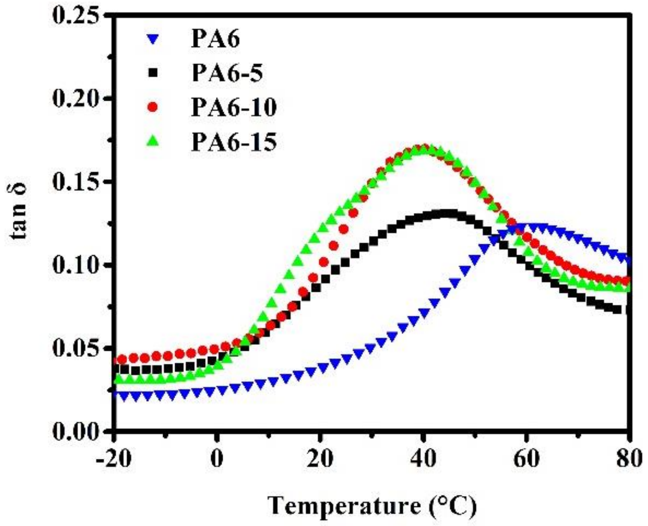

Figure S2. The tan δ curves of PA6 and PA6(BAEA/AA) copolyamides.

S2. Isothermal Crystallization Kinetics

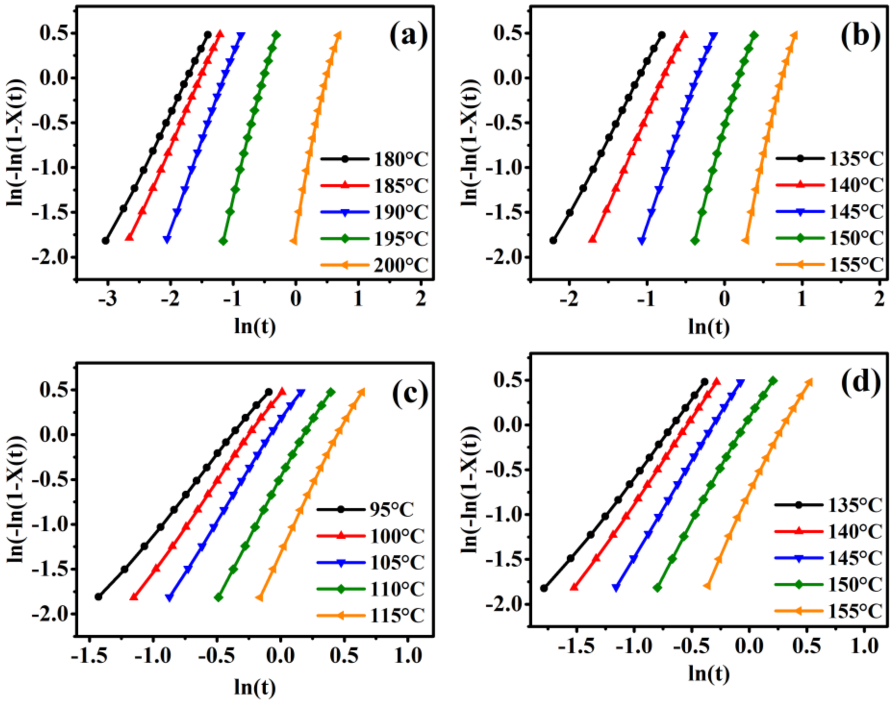

Figure S3. The plots of ln[−ln (1−*X*(*t*))] versus ln(*t*) via the Avrami equation during the different isothermal processes for the (**a**) pure PA6, (**b**) PA6-5, (**c**) PA6-10, and (**d**) PA6-15.

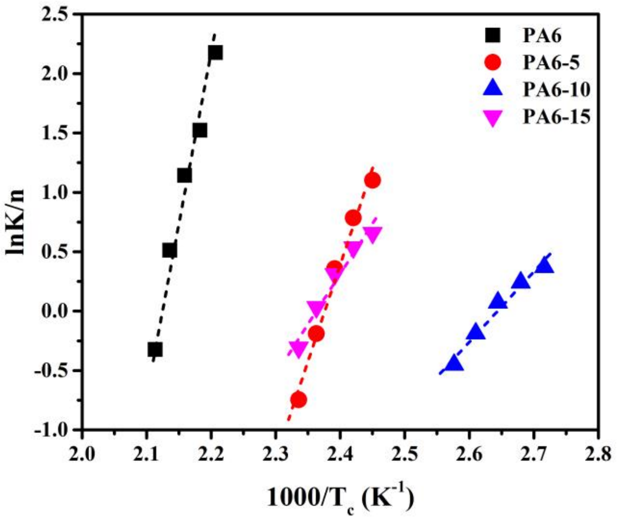

Figure S4. The crystallization activation energy (Δ*E*a) PA6 for copolyamides at different contents of BAEA/AA via the Arrhenius method.

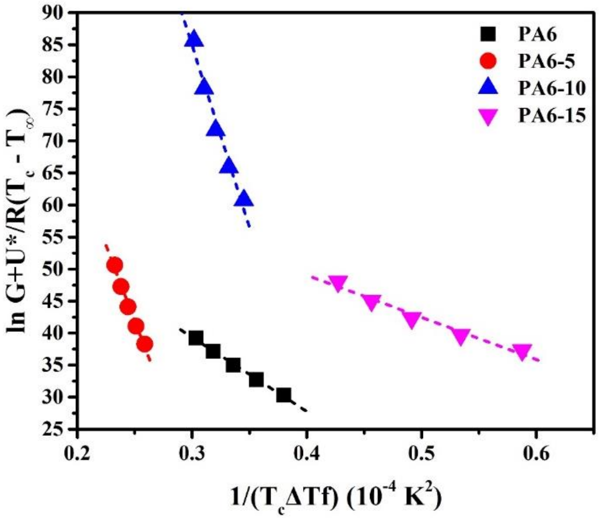

Figure S5. Lauritzen–Hoffman plot for PA6 copolyamides at different contents of BAEA/AA.

References

[1] S.-P. Rwei, T.-F. Way, Y.-H. Chen, Modifying agent, polyamide copolymer synthesized using modifying agent and method for preparing polyamide copolymer, US20190040197, 2018. https://patents.google.com/patent/US20190040197A1/en.

[2] C.-W. Chen, C.-W. Lin, Y.-H. Chen, T.-F. Wei, S.-P. Rwei, R. Sasikumar, The influence of 1,4-cyclohexanedicarboxylic acid on the thermal and mechanical properties of copolyamides, *Polym. Bull.* **2020**, *77*, 235–253. https://doi.org/10.1007/s00289-019-02738-7.

[3] Chen, Y.-H.; Chen, C.-W.; Wei, T.-F.; Rwei, S.-P. Synthesis and characterization of low-temperature polyamide 6 (PA6) copolyamides used as hot melt adhesives and derived from the comonomer of novel aliphatic diamine bis(2-aminoethyl) adipamide and adipic acid. *Int. J. Adhes. Adhes.* **2020**, 102619, doi:10.1016/j.ijadhadh.2020.102619.
